# A Quantitative Assessment of Cerebral Hemodynamic Perturbations Associated with Long R-R Intervals in Atrial Fibrillation: A Pilot-Case-Based Experience

**DOI:** 10.3390/medicina60040531

**Published:** 2024-03-25

**Authors:** Daniela Canova, Silvestro Roatta, Andrea Saglietto, Stefania Scarsoglio, Nefer Roberta Gianotto, Alessandro Piccotti, Gaetano Maria De Ferrari, Luca Ridolfi, Matteo Anselmino

**Affiliations:** 1 Department of Neuroscience, University of Torino, 10125 Torino, Italy; daniela.canova@unito.it (D.C.); silvestro.roatta@unito.it (S.R.); 2 Division of Cardiology, Cardiovascular and Thoracic Department, “Città della Salute e della Scienza” Hospital, 10126 Torino, Italy; andrea.saglietto@live.com; 3 Department of Water Engineering, Politecnico di Torino, 10129 Torino, Italy; stefania.scarsoglio@polito.it; 4 Department of Medical Sciences, University of Torino, 10124 Torino, Italy; nefergianotto@gmail.com (N.R.G.); alessandro.piccotti@edu.unito.it (A.P.); gaetanomaria.deferrari@unito.it (G.M.D.F.); 5 Department of Environment, Land and Infrastructure Engineering, Politecnico di Torino DIATI, 10129 Torino, Italy; luca.ridolfi@polito.it

**Keywords:** cerebral autoregulation, atrial fibrillation, cerebral blood flow, near infrared spectroscopy

## Abstract

*Background and Objectives:* Atrial fibrillation (AF) results in systemic hemodynamic perturbations which impact cerebral circulation, possibly contributing to the development of dementia. However, evidence documenting effects in cerebral perfusion is scarce. The aim of this study is to provide a quantitative characterization of the magnitude and time course of the cerebral hemodynamic response to the short hypotensive events associated with long R-R intervals, as detected by near-infrared spectroscopy (NIRS). *Materials and Methods:* Cerebral NIRS signals and arterial blood pressure were continuously recorded along with an electrocardiogram in twelve patients with AF undergoing elective electrical cardioversion (ECV). The top 0.5–2.5% longest R-R intervals during AF were identified in each patient and used as triggers to carry out the triggered averaging of hemodynamic signals. The average curves were then characterized in terms of the latency, magnitude, and duration of the observed effects, and the possible occurrence of an overshoot was also investigated. *Results:* The triggered averages revealed that long R-R intervals produced a significant drop in diastolic blood pressure (−13.7 ± 6.1 mmHg) associated with an immediate drop in cerebral blood volume (THI: −0.92 ± 0.46%, lasting 1.9 ± 0.8 s), followed by a longer-lasting decrease in cerebral oxygenation (TOI: −0.79 ± 0.37%, lasting 5.2 ± 0.9 s, *p* < 0.01). The recovery of the TOI was generally followed by an overshoot (+1.06 ± 0.12%). These effects were progressively attenuated in response to R-R intervals of a shorter duration. *Conclusions:* Long R-R intervals cause a detectable and consistent cerebral hemodynamic response which concerns both cerebral blood volume and oxygenation and outlasts the duration of the systemic perturbation. These effects are compatible with the activation of dynamic autoregulatory mechanisms in response to the hypotensive stimulus.

## 1. Introduction

Atrial fibrillation (AF)-related perturbations in cerebral circulation are considered to be related to the development of dementia, although there is little direct evidence of such an alteration in brain perfusion.

Modeling studies previously pointed out that in AF, the variability of local hemodynamic variables, such as blood flow and pressure, is increased, particularly at the microcirculatory level. Such variability increases the probability of “extreme events”, e.g., hypo/hypertensive events in capillaries and arterioles, which may cause vascular damage and be causally linked to the development of dementia [1,2,3,4,5]. A recently published study investigated alterations in cerebral circulation in AF patients before and after a intervention using near-infrared spectroscopy (NIRS) (the only available non-invasive methodology allowing one to monitor cerebral microcirculation with adequate time resolution) and provided the first in vivo evidence of AF-related alterations in cerebral hemodynamics [2]. A preliminary analysis of the data showed that variability in arterial blood pressure (ABP) and the tissue hemoglobin index (THI) was high during AF and was reduced by the intervention [2].

The present paper aims to provide a quantification of the hemodynamic effects produced by the extreme events associated with the longest R-R intervals and to extend the analysis to all NIRS variables, particularly the tissue oxygenation index (TOI) [6,7]. In fact, while the THI is an index of hemoglobin concentration and reflects local changes in blood volume, the TOI is directly dependent on tissue perfusion, and its changes can be interpreted in terms of local changes in blood flow, assuming a constant metabolic rate in the tissue [8,9]. To this aim, the triggered-averaging technique was applied, which has the advantage of providing the full time course of the observed phenomenon.

## 2. Materials and Methods

### 2.1. Study Population

Twelve patients (age: 68.9 ± 8.4; 3 females and 9 males) scheduled for electrical cardioversion (ECV) in our Center from January to August 2019 were enrolled in the present analysis. These patients represent a subset of a larger group of patients recruited in a previous study [3] and were selected by eye based on an assessment of good quality and the stability of hemodynamic signals. Out of 20 screened files, 8 were excluded due to the presence of movement artifacts, disturbances introduced by obstructive sleep apnea, or accidental interruptions of the signals. All patients had documented antiarrhythmic drug refractory paroxysmal or persistent AF, defined according to the European Society of Cardiology guidelines [10]. None of them were suffering from diabetes.

Exclusion criteria, in addition to those known to contraindicate ECV, were as follows: an early diagnosis, long-standing persistent or permanent AF; AF in the presence of precipitating factors (e.g., sepsis, acute myocardial ischemia, untreated dysthyroidisms); previous stroke and/or documented brain injury; severe comorbidities (e.g., severe hepatic and renal failure); hemodynamic instability (systolic blood pressure < 90 mmHg, altered consciousness, or signs of reduced peripheral perfusion); and electrolyte abnormalities [11,12,13,14].

This study’s protocol was approved by the local ethics committee and was conducted according to the principles of the Declaration of Helsinki.

All patients provided written informed consent to the procedure. ECV was performed following the Center’s routine and a Day Hospital regimen.

### 2.2. Anticoagulation Protocol and Drug Therapy

All patients received an oral anticoagulant (either a vitamin K antagonist (VKA) or a direct-acting oral anticoagulant (DOAC)) for at least 4 weeks before and after ECV (long-term anticoagulation therapy was then guided by each patient’s thromboembolic risk profile).

For patients on a VKA, the level of anticoagulation was considered adequate if the Index Normalized Ratio (INR) > 2.0 (with an optimal INR range of 2.0–3.0) in the last 4 weeks before ECV; in the case of suboptimal pre-procedural anticoagulation, transesophageal echocardiography (TEE) was performed. All patients on DOAC underwent pre-procedural TEE.

ECV was postponed if thrombi in the left atrium or left atrial appendage were found.

Eleven of the twelve patients were taking antiarrhythmic drugs: Amiodarone (n = 2), Flecainide (6), Sotalol (2), and Sotalol plus Flecainide (1).

All relevant clinical parameters were collected, including the CHA2DS2-VASc score, assessed according to the current European Guidelines on AF [12]. 

### 2.3. Electrical Cardioversion and Monitoring

At admission, every patient underwent a clinical assessment, including a medical history collection, a physical examination, the registration of main cardiovascular parameters (ABP, oxygen saturation (SpO_2_), and heart rate (HR)), an evaluation of ongoing pharmacological therapy, and routine blood tests. 

Once the risk of left atrial thrombus was ruled out, ECV was carried out in a fasting state, under adequate general sedation and in the presence of full equipment for cardiopulmonary resuscitation, according to the actual guidelines [10]. 

Anesthesia was induced using propofol (standard dose 1 mg/kg, titrated according to the patient’s response). 

If necessary, oxygen supplementation was delivered by the anesthesiologist through a bag mask valve until optimal oxygen saturation was achieved. 

Once deep sedation was achieved, ECV was performed by delivering up to three R-wave synchronized direct-current biphasic shocks, adopting a step-up protocol (from 200 to 360 J). ECV was considered successful in the case of sinus rhythm restoration.

**Figure 1:**
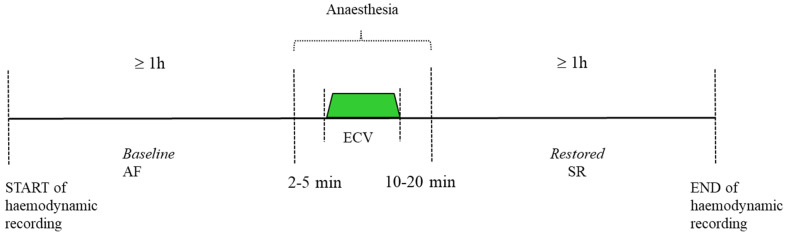
Experimental protocol. AF: atrial fibrillation; SR: sinus rhythm; ECV: electrical cardioversion.

Noninvasive cerebral and systemic hemodynamic monitoring was started at least 1 h before the induction of anesthesia, and continuous recording of all parameters was maintained throughout the ECV procedure until 1 h after full recovery from anesthesia (the patient was usually awake and fully orientated 15–20 min after ECV), according to the experimental protocol in Figure 1.

- Cerebral hemodynamic measurements

Bilateral NIRS monitoring was performed using a two-channel NIRO-200NX monitor (Hamamatsu Photonics K.K. Hamamatsu City, Japan), a device which simultaneously provides Beer–Lambert (BL) conventional spectroscopy and spatially resolved spectroscopy (SRS) variables, allowing one to detect local changes in cerebral tissue oxygenation and blood volume [15].

BL variables express concentration changes in oxyhemoglobin (O_2_Hb), deoxyhemoglobin (HHb), and total hemoglobin (tHb = O_2_Hb + HHb), expressed in μmol/L. The hemoglobin difference signal, Hb(diff) = O_2_Hb − HHb, was also computed as an oxygenation index based on BL variables [16,17].

In addition, SRS variables express the oxyhemoglobin concentration in % (tissue oxygenation index, TOI) and the total hemoglobin concentration in arbitrary units (tissue hemoglobin index, THI). The SRS methodology provides hemodynamic indications which are less affected by superficial extracranial circulation and are thus more specifically related to the deep cerebral tissue compared to BL [15].

For bilateral monitoring, the probes of each channel were placed on the left and right sides of the patient’s forehead, adopting a 4 cm distance between optodes [15].

All NIRS signals were acquired at the NIRO-200NX’s highest sample frequency of 20 Hz, adequate for detecting hemodynamic events associated with single cardiac cycles.

NIRS signals from both channels were separately acquired and digitally transferred to a PC by a proprietary software v1.00E (N200NXOL, Hamamatsu Photonics K.K. Hamamatsu City, Japan).

b. Systemic hemodynamic measurements

Continuous noninvasive measurement of ABP was performed by photo-plethysmography applied to the right third finger (CNAP Monitor 500AT-HD, CNSystems Medizintechnik AG, Graz, Austria); oxygen saturation (SpO_2_) was simultaneously and continuously measured using pulse oximetry applied to the right ring finger, accurately avoiding interference between the two devices (Capnostream^TM^ 20p, Medtronic, Minneapolis, MN, USA). 

Three-lead electrocardiography (ECG) was continuously and simultaneously performed (Dynascope Monitor DS-7100, Fukuda Denshi Co. Ltd., Tokyo, Japan; Defibrillator LIFEPAK 15, Physio-Control, Stryker, Carrigtwohill, Ireland), and a twelve-lead electrocardiogram was printed before and immediately after the ECV to document sinus rhythm restoration (MyCardioPad^XL^, Esaote; Genoa, Italy).

All the above signals were acquired at the same sample frequency of 400 Hz and digitally transferred to a PC by means of the Micro1401-3 multichannel data acquisition system and Spike2 software v.9 (Cambridge Electronic Design Limited, Cambridge, UK). The same software was used for offline processing of the different signals.

### 2.4. Analysis

First, NIRS signals were imported into Spike2 along with the other acquired signals. Proper time alignment was obtained thanks to markers simultaneously provided to both acquisition systems at the beginning and at the end of the recording. The full duration of the recordings for each patient was about 2.5 h.

Triggered averaging was adopted to obtain the average hemodynamic response to the occasionally long R-R intervals that occur in AF, according to the following procedure. In other words, the typical hemodynamic response was obtained as the conditional average of the signal windows corresponding to a threshold exceeded in the R-R interval duration, as described below.

**Figure 2:**
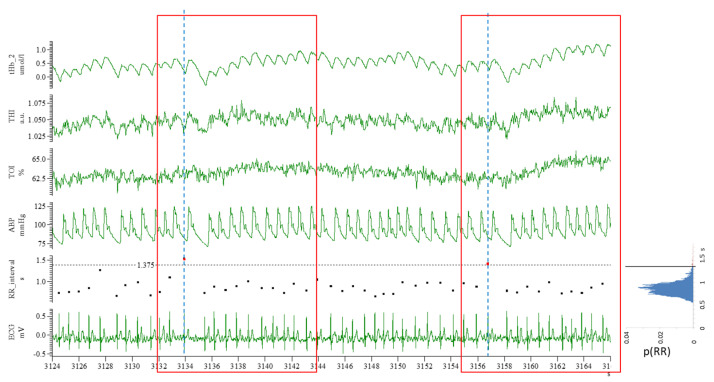
Original recording from a representative patient with atrial fibrillation. From top to bottom: tHb, total hemoglobin; nTHI, normalized Tissue Hemoglobin Index; TOI, Tissue Oxygenation Index; ABP, arterial blood pressure; R-R interval; ECG, electrocardiographic signal. On the right, the probability density function of R-R intervals (p(RR)) shows the frequency distribution of R-R intervals during AF. Long R-R intervals are identified as those exceeding an individually set threshold value (1.38 s in this patient, indicated by the horizontal black line); these are marked as red dots in the RR_interval tracing (two occurrences in the displayed time frame). These events were used as triggers for calculating triggered averages (see text); the width of the triggered average is 12 s, with 2 s of pre-trigger (as indicated by the two red-lined frames for the two selected triggers, further emphasized by the blue vertical dashed lines).

A time series was obtained from the time events corresponding to the R-waves of the ECG. The R-R interval between the current and the next R-wave was calculated (see the RR_interval in Figure 2).

From this series, the frequency distribution of the R-R interval was calculated, as represented in Figure 2, and a threshold (black horizontal line) value was set individually to isolate the top 0.5–2.5% longest R-R intervals (marked as red dots in Figure 2). For each identified R-R interval, a time window of a 12 s width was selected, starting 2 s earlier (pre-trigger) than the first R-wave. The selected windows were then averaged to obtain the response of each signal to long R-R intervals.

To validate the methodology, triggered averages were also calculated from triggers corresponding to R-R intervals of lower values, allowing us to assess the hemodynamic events associated with shorter cardiac cycles during AF. In addition, triggered averages were also calculated for the post-intervention phase under normal sinus rhythm. In this case, all heart beats from a selected interval were used as triggers.

**Figure 3:**
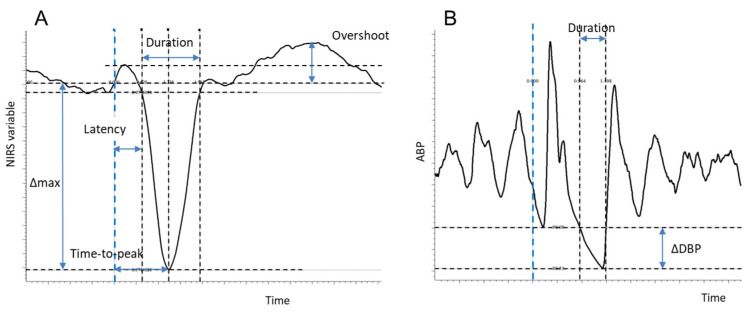
Description of the measurements in NIRS variables (**A**) and in arterial blood pressure (**B**). Blue dashed line: trigger (position of the R-wave).

Average curves of hemodynamic variables were then quantitatively characterized by the measurements described below and schematized in Figure 3A. Note that changes (Δ) with respect to basal (pre-trigger) values are computed in absolute terms for O_2_Hb, HHb, tHb (in μmol/L), and the TOI (in %) and in relative terms for the THI (% of baseline).

Δmax: the maximum change with respect to the basal level, calculated over the 2 s preceding the trigger;

(a) Latency of the response: the time taken to reach 5% of Δmax from the trigger;
(b) Time to peak: the time taken to reach the maximum effect (Δmax) from the trigger;
(c) Duration: the time taken to return to the basal (pre-trigger) level from the beginning of the response (latency);
(d) Overshoot: only for the TOI and Hb(diff), the occurrence of a positive overshoot following the recovery of the initial decrease was detected if the signal exceeded the maximum baseline value and was quantified as the difference between the maximum achieved value and the basal level.

The HHb signal generally presented a diphasic response: a negative phase followed by a positive phase. The first (second) phase was identified as the interval in which the signal decreased and remained below (increased and remained above) 5% of the overall variation (=positive peak − negative peak). The end of the second phase was set to 10 s if a return to baseline was not achieved within the end of the averaging window, which happened in 2 patients. 

Aiming to quantify the drop in pressure associated with the selected long R-R intervals compared to the preceding interval, the following measurements were performed on the average ABP tracing (Figure 3B):

(a) ⊗DBP: the difference between the diastolic blood pressure (DBP) of the cardiac cycle preceding the trigger and the DBP of the cardiac cycle corresponding to the trigger;
(b) Latency: the time taken to reach the diastolic level of the previous cardiac cycle;
(c) Duration: the time taken from the ABP of the trigger’s cardiac cycle to return to the DBP level of the previous cardiac cycle from the beginning of the response (latency).

### 2.5. Statistics

Selective comparisons were made to compare the same measurement (i.e., latency, magnitude, or duration) in different variables (i.e., THI, TOI, O2Hb, etc.) or different measurements in the same variable by means of parametric (Student’s paired t-test, two tails) and non-parametric (Wilcoxon) tests, depending on whether the data were normally distributed or not according to the Shapiro–Wilk test. The SPSS package was used for this purpose.

## 3. Results

Before undergoing ECV, these patients presented the expected highly variable R-R interval of 0.80 ± 0.12 s with an average standard deviation of 0.17 ± 0.04 s, a blood pressure of 100.5 ± 10.3 mmHg, and cerebral oxygenation of 65.9 ± 6.4%. As an example, original recordings from a representative patient are shown in Figure 2, which includes NIRS signals along with ABP and an ECG during AF. The high variability of the R-R interval is clearly visible from the ECG and ABP tracings. Individual R-R intervals are calculated and displayed using dedicated tracings, and the associated frequency histograms are reported on the left. It can be observed that the occurrence of a long R-R interval (i.e., above the individual threshold of 1.375 s) results in a visible perturbation of cerebral hemodynamics, generally more visible in BL than in SRS signals. The thresholds to isolate the long R-R intervals were individually adjusted for each patient; on average, it was set at 1.29 ± 0.18 s (range: 0.93–1.60 s), thus selecting the rightmost 0.5–2.5% of the frequency distribution curve, resulting in a mean frequency of selected (extreme) events of 0.86 ± 0.40 events/min.

**Figure 4:**
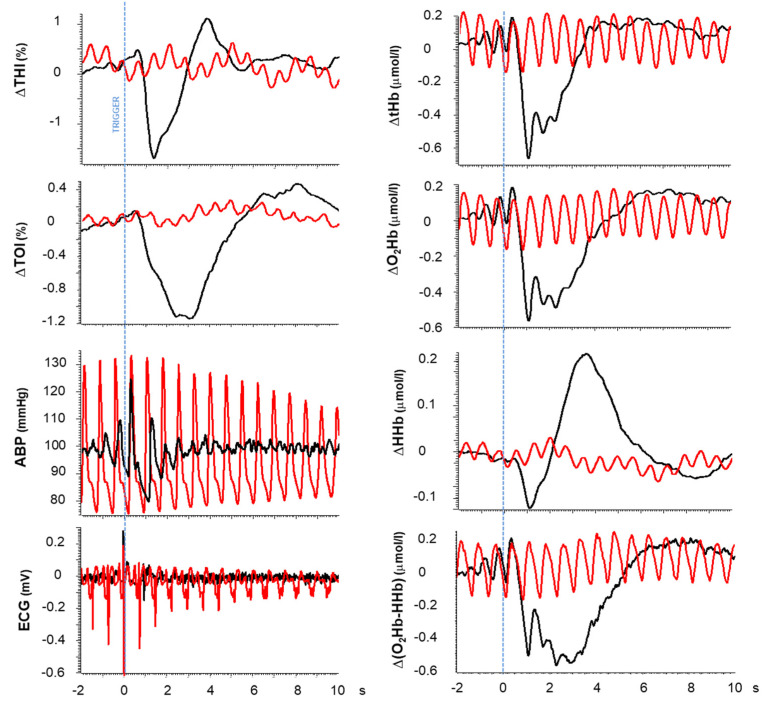
Hemodynamic perturbations caused by long R-R intervals in patient #32. Triggered averages are computed for the different variables based on triggers corresponding to the first R-wave of long R-R intervals (>0.93 s; average: 0.99 ± 0.063 s, n = 62, collected over a 3374 s interval) belonging to the pre-intervention period (black) and consecutive R-R intervals (average: 0.73 ± 0.011 s, n = 122) collected in the post-intervention period (red). For the sake of clarity and a better comparison, the basal (pre-trigger) value was subtracted from each tracing except for ABP and the ECG. THI, normalized Tissue Hemoglobin Index; TOI, Tissue Oxygenation Index; tHb, total hemoglobin; O_2_Hb, oxygenated hemoglobin; HHb, deoxygenated hemoglobin; ABP, arterial blood pressure; ECG, electrocardiographic signal. Note that regular cardiac pulsatility remains visible in all tracings post intervention due to the much lower R-R variability. See the R-R probability density function for this patient in Figure 6A.

**Figure 5:**
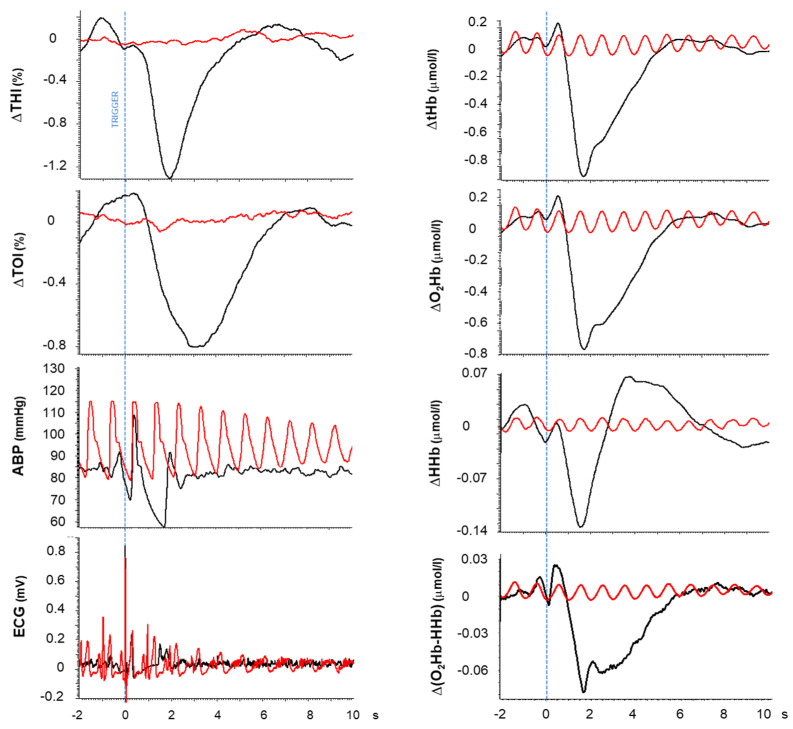
Hemodynamic perturbations caused by long R-R intervals in patient #45. Description as in Figure 4. A selection of long R-R intervals (>1.46 s; average: 1.58 ± 0.11, n = 128, collected over a 4696 s interval) belonging to the pre-intervention period (black) and consecutive R-R intervals (average: 0.97 ± 0.020 s, n = 119) collected in the post-intervention period (red). See the R-R probability density function for this patient in Figure 6B.

The hemodynamic perturbations associated with long R-R intervals during AF were occasionally visible in the original tracings but were consistently evidenced by triggered averaging. Their time course is shown for two representative patients in Figure 4 and Figure 5 (black tracings), while quantifications of the main characteristics such as magnitude and duration are summarized in Table 1 as average measurements over all patients.

**Figure 6:**
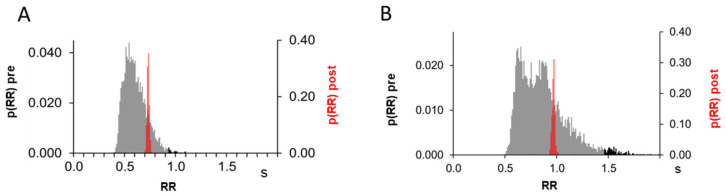
R-R probability density function. R-R probability density functions are shown for the pre- (grey, with selected long intervals in black) and post-intervention (red) periods for the patients described in Figure 4 (**A**) and Figure 5 (**B**).

In all subjects, a sharp and short-lasting drop in cerebral blood volume was detected in the THI (−0.92 ± 0.46%), immediately followed by a decrease in the TOI (−0.79 ± 0.37%) of a considerably longer duration (THI: 1.9 s, TOI: 5.2 s, *p* < 0.01). The time to the peak of the response was also delayed for the TOI compared to the THI (*p* < 0.05).

A transient response to the long RR interval is also detected by the BL variables O_2_Hb and HHb, although with different magnitudes and time courses. O_2_Hb exhibited a prompt and large decrease (0.62 ± 0.20 µmol/L), while in most cases, HHb exhibited a distinct diphasic response of a considerably smaller magnitude (total excursion: 0.20 ± 0.08 µmol/L; *p* < 0.01). In particular, in 11/12 patients, HHb presented an early, short-lasting, and small decrease (0.07 ± 0.04 µmol/L) followed by a larger increase (0.14 ± 0.09 µmol/L, *p* < 0.01) and duration (*p* < 0.01); in one patient, HHb only presented the latter increasing phase. Given the relatively small HHb contribution, the time course of tHb closely reflected the O_2_Hb changes. Compared to the THI, the tHb exhibited a significantly slower return to baseline (*p* < 0.01).

Further attention was devoted to changes in tissue oxygenation given its possible interpretation as an index of blood flow (see discussion). In particular, we noticed that a positive overshoot was frequently exhibited by the TOI following the return to baseline in 10/12 patients (on average, 1.06 ± 0.12%). Interestingly, this overshoot was detected in only 2/12 patients in the hemoglobin difference signal.

**Figure 7:**
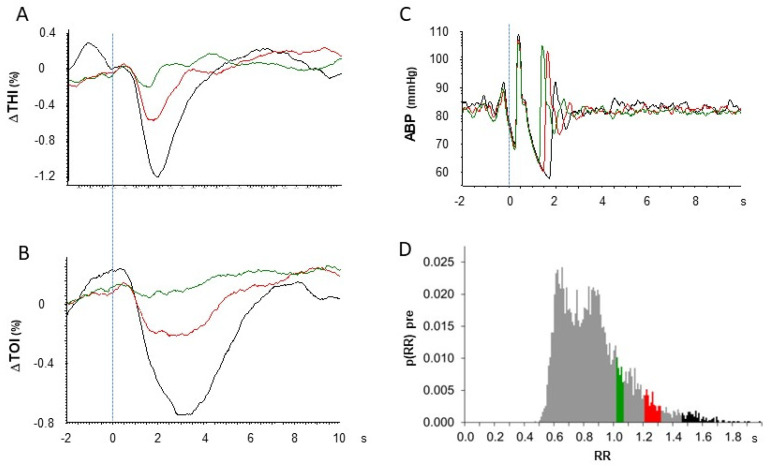
Methodological validation. Triggered averages for the THI (**A**), TOI (**B**), and ABP (**C**) are calculated using R-waves related to R-R intervals of different durations as trigger: R-R > 1.46 (black line, n = 128, average: 1.58 s), 1.21 < R-R < 1.31 (red line, n = 184, average: 1.25 s), and 1.02 < R-R < 1.06 (green line, n = 201, average: 1.04 s) for the same patient described in Figure 4. The trigger is represented by the dashed line. nTHI, normalized Tissue Hemoglobin Index; TOI, Tissue Oxygenation Index; ABP, arterial blood pressure. Time 0 is the time of the R-wave at the beginning of the selected R-R interval (trigger). At the bottom right (**D**), the probability density function shows the distribution of R-R intervals in this patient; the selected ranges for R-R interval duration are highlighted with the corresponding colors.

Average tracings were also collected from ABP and the ECG. As expected, the ABP signal exhibited a consistent drop in diastolic blood pressure during long RR intervals. This was quantified by a comparison with the diastolic pressure in the preceding cardiac cycle (Figure 3B). The magnitude and duration of the diastolic pressure drop were, on average, 13.7 ± 6.1 mmHg and 0.64 ± 0.13 s, respectively, while the mean ABP returned to its basal value within 3 s. The ECG tracing was included in the figure to clarify the role of the R-wave as trigger for the averaging, indicated by the blue dashed line and serving as reference for the time axis (Figure 4, Figure 5 and Figure 7).

The above-described hemodynamic effects were no longer detected after the sinus rhythm was re-established, and the triggered average was then driven by the R-waves of regular heart beats, which occurred at a fairly constant heart rate. It can be observed from the red tracings in Figure 4 and Figure 5 that the hemodynamic changes associated with regular heart beats were simple oscillations of much smaller magnitudes and durations (equal to the duration of the cardiac cycle). Due to the high regularity of the cardiac rhythm, these oscillations were time-aligned in all triggered occurrences and were not erased by the triggered averaging; on the contrary, they exhibited an almost constant magnitude over the 10 s post-trigger interval. Note that oscillations related to the cardiac rhythm were often present in BL parameters but only occasionally in SRS (e.g., the patient in Figure 3 but not the patient in Figure 4). Irrespective of this, the response to long R-R intervals was always clearly detected by triggered averaging.

To further verify that the observed effects were causally related to long R-R intervals, we investigated the dependence of the magnitude of the hemodynamic effect on the duration of the R-R interval. To this aim, triggered averages were recalculated for RR intervals of different durations that were selected within narrow ranges from the pre-intervention phase. The results are presented in Figure 7 for the THI, TOI, and ABP in one patient and show that the magnitude of the hemodynamic change progressively decreased with the duration of RR intervals; the maximum effect is observed when R-R > 1.46 s (black line) and progressively reduces when the duration is reduced to 1.21 < R-R < 1.31 s (red line) and 1.02 < R-R < 1.06 s (green line) until its complete disappearance if all R waves are considered, either pre- or post-intervention. Similar trends are observed in the THI and TOI and are consistent with the progressively reduced drop in ABP (Figure 7C). The dependence of hemodynamic responses on the R-R duration was investigated in other two patients, obtaining equivalent results.

## 4. Discussion

For the first time, a quantitative analysis of the cerebral hemodynamic effects produced by the hemodynamic perturbations associated with long R-R intervals has been performed in AF patients by means of R-wave triggered averaging. The results evidenced that (i) long R-R intervals are “extreme events” that produce transient but marked drops in blood pressure and corresponding transient decreases in local cerebral blood volume and oxygenation; (ii) RR intervals of a shorter duration result in pressure drops and cerebral hemodynamic effects of progressively lower magnitudes, thus confirming the causal relationship between the extreme event and the hemodynamic effect; (iii) the TOI decrease appears to be slower than that of the THI, lasting about 7–10 s, and is followed by a phase of overshoot; (iv) a similar time course is exhibited by BL NIRS variables, with some differences that can be attributed to extracranial contamination.

### 4.1. Effect on Cerebral Hemodynamics

In a previous study, Saglietto et al. [18] already evidenced that in individual cardiac cycles, the pulse pressure is proportional to the duration of the RR interval. We here focused our attention on this systemic perturbation and quantified the typical extent (13.7 mmHg) and duration (0.64 s) of the diastolic pressure drop. In addition, we noticed that the actual recovery of mean ABP requires a longer time, in the order of about 3 s, as evidenced by the triggered averaging (Figure 4 and Figure 5). This is the duration of the actual perturbation which propagates to cerebral circulation, producing drops in transmural pressure and cerebral perfusion pressure and resulting in complex cerebral hemodynamic changes. In this respect, the early and relatively short-lasting drop in the THI, which closely mimics the local drop in arterial blood pressure (see Figure 4 and Figure 5), likely reflects the passive resizing of blood vessels due to a decrease in transmural pressure. This partial “deflation”, presumably concerning the large cerebral blood arteries (which are more exposed to the blood pressure drop) in particular, depends on the compliance of the arterial network and is likely to account for the *Windkessel* effect, which may attenuate the ensuing changes in cerebral blood flow. In fact, it was recently proposed that this effect may almost completely compensate for the transient drops in ABP as produced by lower body negative pressure [19], thigh-cuff deflation, and the sitting-to-standing transition [20]. However, this hypothesis is refuted by the present data, in which a drop in tissue oxygenation is consistently observed. 

The drop in tissue oxygenation exhibits a longer latency and slower recovery than the THI. Tissue oxygenation depends on the balance between metabolism and perfusion. Consequently, in conditions of putatively constant brain metabolism, changes in the TOI can be interpreted in terms of changes in blood flow [9]. On this basis, the present drop in the TOI reflects a drop in blood flow caused by a drop in perfusion pressure. Notably, the return to the basal level takes more than 5 s, which likely is the time required for the extra amount of desaturated Hb to be washed out from the venous compartment of the sample volume. This interpretation is consistent with the early increase in deoxygenated hemoglobin, as indicated by HHb.

From a quantitative point of view, the magnitude of the peak effects appears rather small, about a 1% drop in blood volume and about a 1% drop in tissue oxygenation (see the peak effects of the THI and TOI, respectively, in Table 1). As for the volume drop, we have to remember that cerebral circulation is constrained in the virtually constant intracranial volume. Moreover, the NIRS measurement reflects average changes occurring in the whole sample volume, which includes arteries, arterioles, capillaries, and veins. The change in blood volume is likely to be exhibited only by the upstream arterial/arteriolar compartment where the transmural pressure change is larger. Consequently, a small decrease in the whole blood volume may reflect a larger change at the arterial level. Along the same line, the 1% drop in Hb saturation over the whole sample volume is likely related to a larger decrease at the capillary level. In addition, this apparently small hemodynamic effect appears to be large enough to elicit an active vascular response by the cerebral tissue, as discussed below.

### 4.2. The Active Cerebrovascular Response to the Hypotensive Insult

Several observations support the occurrence of a cerebrovascular response to the hypotensive/hypoperfusive perturbations associated with long RR intervals. First of all, a significant overshoot is exhibited by the TOI after the initial drop. This necessarily results from an increase in blood flow which, in the absence of an increase in ABP, can only descend from an active decrease in cerebrovascular resistance.

Additional support comes from the comparison of SRS and BL variables, with the latter being heavily affected by the extracranial compartment, which is characterized by a less prominent autoregulatory capacity than the deep cerebral compartments. In particular, it can be observed that (i) the difference O_2_Hb-HHb, which provides an index of tissue oxygenation based on BL variables, contrary to the TOI, does not exhibit a significant overshoot after recovering from the initial drop; and (ii) the blood volume indicator, tHb, exhibits a significant slower recovery than the THI. Both observations support the idea that the active vascular reaction is indeed a characteristic of the intracranial compartment and that it takes place within the 10 s lasting temporal window examined in this study. This vascular response may possibly include a myogenic dilatation elicited by the drop in local transmural pressure, as well as a metabolic dilatatory response triggered by the transient decreases in blood flow and oxygenation [21,22].

In fact, the hypotensive insult associated with the long RR interval is not dissimilar to stimuli delivered to investigate the dynamic cerebral autoregulation, as originally proposed by Aaslid et al. [23], namely, the brief (1.3 s) occlusion of the common carotid artery, selectively affecting one hemisphere, and the sudden release of thigh cuffs (previously inflated at 200 mmHg for 2 min), which produces a transient drop in ABP (about 20 mmHg) which recovers over more than 10 s. 

By monitoring blood velocity in the middle cerebral artery (V_MCA_) using transcranial Doppler sonography, they showed that functional autoregulation is connoted by a prompt hyperemia immediately developing after the release of common carotid artery occlusion and an early recovery of the V_MCA_ compared to ABP after thigh cuff release. Subsequent studies investigated the response to thigh cuff release with NIRS [24] and diffuse correlation spectroscopy, which provides a semiquantitative measure of cortical cerebral blood flow [25]. They confirmed the occurrence of an early dynamic autoregulation, reporting transient and small decreases in blood flow (−4%) and blood volume (−2%), a decrease in oxygenation (resulting from decreased O_2_Hb and increased HHb), and a prompt decrease in cerebrovascular resistance.

Although thigh cuff release generates a relatively long hypotensive stimulus (>10 s), the first part of the ensuing hemodynamic response is comparable to the response to long RR intervals presently observed in terms of the transient decreases in and early recovery of blood volume and oxygenation, which confirms the occurrence of prompt dynamic cerebral autoregulation.

However, it can be hypothesized that in the long run, these rebound effects and their repetitive occurrence in AF may be harmful and may have a role in the development of functional impairment possibly leading to dementia.

### 4.3. Limitations

A major limitation of the standard modified-BL methodology is the fact that the contribution of superficial tissue layers (skin) to the hemodynamic measurement cannot be discriminated from deeper ones (brain and muscle). However, this limitation was overcome by the SRS methodology. With SRS NIRS, a differential signal is calculated from the backscattered light collected from three close photodetectors, a process that attenuates the common input mainly derived from superficial layers and preserves contributions from deeper tissues [26]. The capacity of SRS to focus the measurement on deeper tissue layers has been now documented in the brain during different vegetative [7,15] and pharmacological tests [27,28]. On this basis, the changes detected in the SRS variables TOI and THI are considered to specifically indicate cerebral hemodynamic changes, while the indications provided by BL variables (HHb, O_2_Hb, and tHb) need to be interpreted more cautiously, taking into account that they are also affected by extracranial hemodynamics.

A second limitation is the limited sample size. However, the hemodynamic effect was consistent among subjects, which allowed us to obtain a statistically sound quantification of the phenomenon. 

Finally, NIRS only provides an indirect indication of blood flow changes. Further studies adopting transcranial Doppler flowmetry or functional magnetic resonance imaging may confirm the observations of the present study.

## 5. Conclusions

The present quantitative analysis of cerebral hemodynamic effects produced by the hemodynamic perturbations associated with long R-R intervals in AF patients highlights that long R-R intervals cause a detectable and consistent cerebral hemodynamic response which concerns both cerebral blood volume and oxygenation and outlasts the duration of the systemic perturbation. 

## Figures and Tables

**Table 1:** Mean effects in NIRS variables. nTHI, normalized Tissue Hemoglobin Index; TOI, Tissue Oxygenation Index; tHb, total hemoglobin; O_2_Hb, oxygenated hemoglobin; HHb, deoxygenated hemoglobin; ⊗_max_, the maximum change from baseline; latency, the start of the response from the trigger; time to peak, the time to reach the maximum effect from the trigger; duration, the duration of the effect. * With regard to HHb_decrease_, n = 11 due to the absence of the first phase in one response.

	THI (%)		TOI (%)		tHb (mmol/L)		O_2_Hb (mmol/L)		HHb_decrease_ * (mmol/L)		HHb_increase_ (mmol/L)	
	Mean	St Dev	Mean	St Dev	Mean	St Dev	Mean	St Dev	Mean	St Dev	Mean	St Dev
D_max_	−0.92	0.46	−0.79	0.37	−0.68	0.22	−0.62	0.20	−0.07	0.04	0.14	0.09
latency (s)	0.68	0.13	0.81	0.28	0.73	0.10	0.74	0.11	0.69	0.16	2.06	0.62
time to peak (s)	1.41	0.24	2.71	0.47	1.43	0.21	1.52	0.36	1.24	0.25	3.49	0.56
duration (s)	1.86	0.80	5.17	0.94	3.91	0.89	4.90	1.03	1.20	0.56	5.66	2.12

## Data Availability

The data supporting the findings of this study are available from the corresponding author upon reasonable request.

## References

[1] Saglietto A., Scarsoglio S., Ridolfi L., Gaita F., Anselmino M. (2019). Higher ventricular rahte during atrial fibrillation relates to increased cerebral hypoperfusions and hypertensive events. Sci. Rep..

[2] Saglietto A., Scarsoglio S., Canova D., Roatta S., Gianotto N., Piccotti A., Franzin S., Gaita F., De Ferrari G.M., Ridolfi L. (2021). Increased beat-to-beat variability of cerebral microcirculatory perfusion during atrial fibrillation: A near-infrared spectroscopy study. EP Eur..

[3] Zhai Y., Hu F., Yuan L., Ye X., Shi W., Yang R., Cao Y., Sun J., He J., Xu F. (2024). Atrial fibrillation increases the risk of all-cause dementia, Alzheimer’s disease, and vascular dementia: A cohort study of 373, 415 participants in the UK Biobank. J. Affect. Disord..

[4] Carbone G., Ercolano E., Bencivenga L., Palaia M.E., Scognamiglio F., Rengo G., Femminella G.D. (2024). Atrial Fibrillation and Dementia: Focus on Shared Pathophysiological Mechanisms and Therapeutic Implications. J. Am. Med. Dir. Assoc..

[5] Calvert P., Gupta D., Lip G.Y.H. (2023). The neurocognitive effects of atrial fibrillation: Benefits of the ABC pathway. Eur. Heart J.-Cardiovasc. Pharmacother..

[6] Al-Rawi P.G., Kirkpatrick P.J. (2006). Tissue oxygen index: Thresholds for cerebral ischemia using near-infrared spectroscopy. Stroke.

[7] Quaresima V., Sacco S., Totaro R., Ferrari M. (2000). Noninvasive measurement of cerebral hemoglobin oxygen saturation using two near infrared spectroscopy approaches. J. Biomed. Opt..

[8] Fadel P.J., Keller D.M., Watanabe H., Raven P.B., Thomas G.D. (2004). Noninvasive assessment of sympathetic vasoconstriction in human and rodent skeletal muscle using near-infrared spectroscopy and Doppler ultrasound. J. Appl. Physiol..

[9] Fantini S., Sassaroli A., Tgavalekos K.T., Kornbluth J. (2016). Cerebral blood flow and autoregulation: Current measurement techniques and prospects for noninvasive optical methods. Neurophotonics.

[10] Camm A.J., Kirchhof P., Lip G.Y.H., Schotten U., Savelieva I., Ernst S., Van Gelder I.C., Al-Attar N., Hindricks G., Prendergast B. (2010). Guidelines for the management of atrial fibrillation. Europace.

[11] Toso E., Blandino A., Sardi D., Battaglia A., Garberoglio L., Miceli S., Azzaro G., Capello A.L., Gaita F. (2012). Electrical cardioversion of persistent atrial fibrillation: Acute and long-term results stratified according to arrhythmia duration. Pacing Clin. Electrophysiol..

[12] Wutzler A., Nee J., Boldt L.H., Kühnle Y., Gräser S., Schröder T., Haverkamp W., Storm C. (2014). Improvement of cerebral oxygen saturation after successful electrical cardioversion of atrial fibrillation. Europace.

[13] Barrett O.S.H., Macdonald S.P.J., Playford D.A. (2015). Near-infrared spectroscopy-based microcirculatory assessment in acute atrial fibrillation. Anaesth. Intensive Care.

[14] Toso E., Iannaccone M., Caponi D., Rotondi F., Santoro A., Gallo C., Scaglione M., Gaita F. (2017). Does antiarrhythmic drugs premedication improve electrical cardioversion success in persistent atrial fibrillation?. J. Electrocardiol..

[15] Canova D., Roatta S., Bosone D., Micieli G. (2011). Inconsistent detection of changes in cerebral blood volume by near infrared spectroscopy in standard clinical tests. J. Appl. Physiol..

[16] Wagner B.P., Pfenninger J. (2002). Dynamic cerebral autoregulatory response to blood pressure rise measured by near-infrared spectroscopy and intracranial pressure. Crit. Care Med..

[17] Delpy D.T., Cope M. (1997). Quantification in tissue near-infrared spectroscopy. Philos. Trans. R. Soc. B Biol. Sci..

[18] Saglietto A., Scarsoglio S., Canova D., De Ferrari G.M., Ridolfi L., Anselmino M. (2023). Beat-to-beat finger photoplethysmography in atrial fibrillation patients undergoing electrical cardioversion. Sci. Rep..

[19] Shoemaker L.N., Milej D., Sajid A., Mistry J., Lawrence K.S., Shoemaker J.K. (2023). Characterization of cerebral macro- and microvascular hemodynamics during transient hypotension. J. Appl. Physiol..

[20] Tzeng Y.C., MacRae B.A., Ainslie P.N., Chan G.S.H. (2014). Fundamental relationships between blood pressure and cerebral blood flow in humans. J. Appl. Physiol..

[21] Davis M.J. (2012). Perspective: Physiological role(s) of the vascular myogenic response. Microcirculation.

[22] Brassard P., Roy M.A., Burma J.S., Labrecque L., Smirl J.D. (2023). Quantification of dynamic cerebral autoregulation: Welcome to the jungle!. Clin. Auton. Res..

[23] Aaslid R., Newell D.W., Stooss R., Sorteberg W., Lindegaard K.F. (1991). Assessment of cerebral autoregulation dynamics from simultaneous arterial and venous transcranial Doppler recordings in humans. Stroke.

[24] Kainerstorfer J.M., Sassaroli A., Tgavalekos K.T., Fantini S. (2015). Cerebral autoregulation in the microvasculature measured with near-infrared spectroscopy. J. Cereb. Blood Flow. Metab..

[25] Parthasarathy A.B., Gannon K.P., Baker W.B., Favilla C.G., Balu R., Kasner S.E., Yodh A.G., Detre J.A., Mullen M.T. (2018). Dynamic autoregulation of cerebral blood flow measured non-invasively with fast diffuse correlation spectroscopy. J. Cereb. Blood Flow. Metab..

[26] Scholkmann F., Kleiser S., Metz A.J., Zimmermann R., Mata Pavia J., Wolf U., Wolf M. (2014). A review on continuous wave functional near-infrared spectroscopy and imaging instrumentation and methodology. Neuroimage.

[27] Moerman A.T., Vandenheuvel M., Tuybens P.J., Van Gompel C., De Hert S.G. (2022). Incongruous effect of phenylephrine on changes in cerebral blood volume measured by near-infrared spectroscopy (NIRS) indicating extracranial contamination. J. Clin. Monit. Comput..

[28] Grassi B., Quaresima V. (2016). Near-infrared spectroscopy and skeletal muscle oxidative function in vivo in health and disease: A review from an exercise physiology perspective. J. Biomed. Opt..

